# Systematic Review with Meta-analysis: Association of Helicobacter pylori Infection with Esophageal Cancer

**DOI:** 10.1155/2019/1953497

**Published:** 2019-12-01

**Authors:** Huiqin Gao, Lunan Li, Chenjing Zhang, Jiangfeng Tu, Xiaoge Geng, Jingya Wang, Xiaolu Zhou, Jiyong Jing, Wensheng Pan

**Affiliations:** ^1^Department of Gastroenterology, Zhejiang Provincial People's Hospital, People's Hospital of Hangzhou Medical College, Hangzhou, Zhejiang, China; ^2^Bengbu Medical College, Bengbu, Anhui, China; ^3^Zhejiang Provincial People's Hospital, People's Hospital of Hangzhou Medical College, Hangzhou, Zhejiang, China

## Abstract

**Background:**

Helicobacter pylori is an important carcinogenic factor in gastric cancer. Studies have shown that Helicobacter pylori infection is inversely associated with certain diseases such as esophageal cancer and whose infection appears to have a “protective effect.” At present, the relationship between Helicobacter pylori infection and esophageal cancer remains controversial. This study was designed to investigate the relationship between Helicobacter pylori infection and the risk of esophageal cancer in different regions and ethnicities.

**Methods:**

Systematic search of the articles on the relationship between Helicobacter pylori infection and esophageal cancer from the database with the duration time up to December 2018. This systematic review was performed under the MOOSE guidelines.

**Results:**

This meta-analysis included 35 studies with 345,886 patients enrolled. There was no significant correlation between Helicobacter pylori infection and esophageal squamous cell carcinoma in the general population (OR: 0.84; 95% CI: 0.64-1.09/OR: 0.74; 95% CI: 0.54-0.97). However, a significant correlation was found in the Middle East (OR: 0.34; 95% CI: 0.22-0.52/95% CI: 0.26-0.44). There was no significant difference in the prevalence of Helicobacter pylori between the case group and the control group in esophageal adenocarcinoma (8.87% vs. 9.67%). The pooled OR was 0.55 (95% CI: 0.43-0.70) or 0.23 (95% CI: 0.15-0.36). When grouped by match or not, the pooled OR of the nonmatching group and the matching group was 0.48/0.21 (95% CI: 0.36-0.65/95% CI: 0.13-0.36) and 0.73/0.71 (95% CI: 0.57-0.92/95% CI: 0.60-0.84), respectively.

**Conclusion:**

In the general populations, no significant association was found between Helicobacter pylori infection and the risk of esophageal squamous cell carcinoma. However, lower risk was found in the Middle East. Helicobacter pylori infection may reduce the risk of esophageal adenocarcinoma, but such “protection effect” may be overestimated.

## 1. Introduction

Esophageal cancer ranks the eighth in the world's cancer incidence and the sixth in the global cancer death cause [1]. There are two major histological subtypes of the esophagus: esophageal squamous cell carcinoma (ESCC) and esophageal adenocarcinoma (EAC). ESCC and EAC have different geographic and demographic models. ESCC has a high incidence in many developing countries. The most important risk factors in Western countries are smoking and habitual consumption of alcohol. In developed countries such as North America, Australia, and Europe, esophageal adenocarcinoma has become the main subtype of esophageal cancer; its major risk factors include chronic gastroesophageal reflux disease, obesity, and smoking [2–4].

Helicobacter pylori is a common bacterium in the upper digestive tract, which infects about half of the world [5]. Marshall and Warren first reported the cultivation of Helicobacter pylori from human gastric mucosa in 1983 [6]. The International Agency for Research on Cancer and the World Health Organization believed that Helicobacter pylori is a carcinogen of gastric cancer [7]. However, some studies have shown that Helicobacter pylori infection is negatively correlated with some diseases [8–11]. Helicobacter pylori infection appeared to have a “protective effect.” Since the 20th century, the prevalence of Helicobacter pylori has declined in Western countries; the incidence of esophageal cancer has subsequently increased. Although the previous meta-analysis has systematically illustrated on the relationship between them, there has been controversy [12–15]. At present, the relationship between Helicobacter pylori and esophageal squamous cell carcinoma has not been clearly explained; the evidence of its protective or harmful effects on esophageal adenocarcinoma is still contradictory. In recent years, articles on the relationship between Helicobacter pylori and esophageal cancer have been published in succession; new data can be used to further analyze the relationship between Helicobacter pylori and esophageal cancer. Moreover, whether there are different relations between different regions and ethnicities has not been specifically explained. Therefore, we did a meta-analysis to explore the relationship between Helicobacter pylori infection and the risk of esophageal cancer in different regions and ethnicities.

## 2. Methods

The data of this meta-analysis were collected based on the Meta-analysis of Observational Studies in Epidemiology (MOOSE) statements [16] ([Supplementary-material supplementary-material-1]).

### 2.1. Data Source and Search Strategy

All articles and abstracts published up to December 2018 were systematically searched in Embase and PubMed using MeSH terms and free words. Some of the database search MeSH terms are the following: “Esophageal Neoplasms”, “Esophageal squamous cell carcinoma”, “Adenocarcinoma of Esophagus”, and “Helicobacter pylori”. Comprehensive search terms are listed in the appendix. Besides, manual searches were conducted to ensure that all articles were related to our subject. Other sources are from related articles mentioned in the previous related meta-analysis [17–20]. There are no predetermined study design types, language restrictions, or publication years.

### 2.2. Study Selection

The two authors (HQ Gao and LN Li) independently selected articles to be included and conducted critical assessments. Discrepancies were resolved by reaching a consensus with the senior author (JY Jing). Eligible studies were selected according to the following inclusion criteria: (1) they are studies on the relationship between Helicobacter pylori infection and esophageal cancer, (2) the incidence of esophageal cancer in the control and case groups can be extracted when exposed to or not exposed to Helicobacter pylori, (3) the odds ratio (OR) (case-control study) or relative risk (RR) (cohort study) and 95% confidence interval (95% CI) can be directly or indirectly calculated, and (4) the full text can be obtained. The exclusion criteria are (1) unobtainable full text, (2) the repeated study of the same population sample, and (3) the secondary data analysis literature such as review or meta-analysis.

### 2.3. Data Extraction

Data extraction was independently completed by the two researchers (HQ Gao and LN Li). First of all, after the thorough review of the title and abstract, obtain the full-text literature which meets the inclusion criteria. Two sides discuss for the controversial literature and seek the third senior researcher (JY Jing) to make a final decision if controversies were meet. The following data were extracted from each article: first author's name, year of publication, study country, ethnicity, study type, study subject, Helicobacter pylori test method, and number of control and case groups, as well as related measurement indicators (OR or RR and 95% CI).

### 2.4. Quality Assessment

Two authors (HQ Gao and LN Li) independently evaluated the quality of the articles. Discrepancies were resolved by reaching a consensus with the senior author (JY Jing). The quality of the literature was evaluated using the Newcastle-Ottawa Scale (NOS) [21]. The scale was evaluated from study population selection (4 points), intergroup comparability (2 points), and exposure or outcome evaluation (3 points). The total score is 9 points, with higher scores indicating better methodological quality.

### 2.5. Risk of Bias Assessment

The two authors (HQ Gao and LN Li) performed the risk assessment of the included studies. Any disputes or inconsistencies were discussed in the group to achieve a consistent result. The risk of bias was evaluated by the ROBINS-I (Risk Of Bias In Nonrandomized Studies—of Interventions) tool [22]. Based on the risk of bias of seven different domains, the overall bias risk for each outcome and study was estimated. It is divided into three parts: preintervention (bias due to confounding, bias in selection of participants into the study), at intervention (bias in classification of interventions), and postintervention (bias due to deviations from intended interventions, bias due to missing data, bias in measurement of outcomes, and bias in selection of the reported result).

### 2.6. Statistical Analysis

The relationship between Helicobacter pylori and esophageal cancer was summarized by the binary method. OR and 95% CI were calculated for each study. To investigate the sources of heterogeneity, we carried out the following tests: heterogeneity tests, subgroup analysis, metaregression analysis, and sensitivity analysis. The heterogeneity of each study was statistically analyzed by *Q* test and *I*^2^ test. When *I*^2^ was 0–40%, it means littler no heterogeneity, 30–60% means moderate heterogeneity, 50–90% indicates substantial heterogeneity, and 75-100% indicates considerable heterogeneity [23]. If there is moderate heterogeneity or above, we use the Hartung-Knapp-Sidik-Jonkman (HKSJ) method for further statistics [24]. The random effects model is used to pool the analysis [25], and subgroup analysis is carried out according to the factors that may cause heterogeneity (ethnicity, study area, study type, study object, Helicobacter pylori detection method, and whether the study population of control group and case group is matched by gender, age, and race). Subsequently, restricted maximum likelihood-based random effects metaregression analyses were carried out to evaluate potential heterogeneous factors. Univariate metaregression analysis was conducted first, after which the variables that were significant at the 0.1 level were entered into the multivariable model. Sensitivity analysis verifies the stability of the results by eliminating each study one by one. Assessment of potential publication bias was made using Harbord weighted linear regression and funnel plots. Data collation and analysis were performed using Stata13.1 software and RStudio software.

## 3. Results

### 3.1. Characteristics of Studies

**Figure 1:**
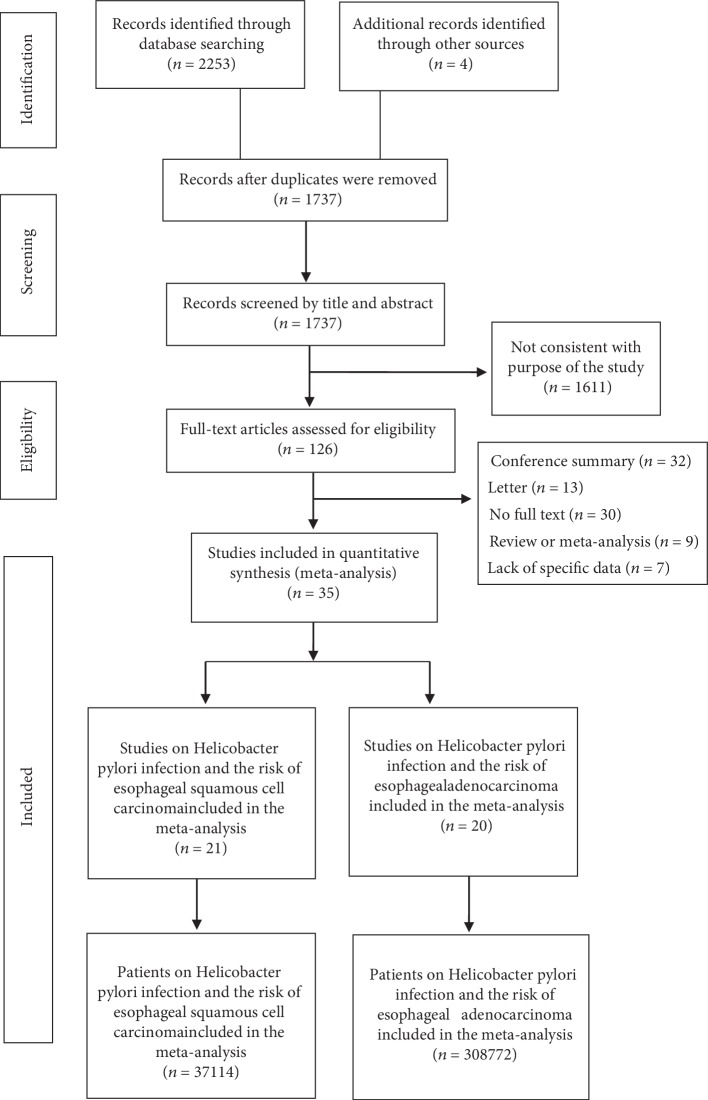
Flowchart showing selection of publications for review.

According to the inclusion and exclusion criteria, a total of 35 studies were included in our study [17–20, 26–56]. A flow diagram for our systematic review is shown in Figure 1.  Table 1 summarized the baseline characteristics of the included studies. Twelve studies from North America [17–20, 28, 40, 46, 48, 50, 54–56], ten studies from Europe [26, 32, 33, 36, 39, 42, 43, 49, 52, 53], eight studies from East Asia [27, 31, 37, 38, 44, 45, 47, 51], four studies from the Middle East [29, 30, 34, 41], and one study from Oceania [35]. Regarding the study type, thirty-three of them are case-control studies [17–20, 27–30, 32–56] and two of them are cohort studies [26, 31].  Table 2(a) summarized the data of Helicobacter pylori infection in esophageal squamous cell carcinoma, with a total of 37,114 patients, including 2,063 patients in the case group and 35,051 patients in the control group.  Table 2(b) contains the data of esophageal adenocarcinoma, with a total of 308,772 patients, including 7,687 patients in the case group and 301,085 patients in the control group. Furthermore, 14 studies involved esophageal adenocarcinoma [18, 19, 28, 39–42, 46, 48, 50, 53–56], 15 studies involved esophageal squamous cell carcinoma [17, 27, 29–34, 36–38, 44, 45, 47, 51], and 6 studies involved both types [20, 26, 35, 43, 49, 52]. According to the Newcastle-Ottawa Scale, sixteen studies were ranked as very good [17, 19, 20, 26, 29, 32, 33, 35, 39, 40, 43, 44, 48–50, 56], seventeen as good [18, 28, 31, 34, 36–38, 41, 42, 45–47, 51–55], and two as satisfactory [27, 30] ([Supplementary-material supplementary-material-1]).

### 3.2. Risk of Bias within Studies

According to the ROBINS-I tool, four studies had a serious risk of bias [19, 34, 37, 46], 26 studies had a moderate risk of bias [17, 18, 20, 26, 27, 29–31, 33, 35, 38–43, 45, 47–51, 53–56], and five of the other studies had a low risk of bias [28, 32, 36, 44, 52] ([Supplementary-material supplementary-material-1]).

### 3.3. Helicobacter pylori Infection and the Risk of Esophageal Squamous Cell Carcinoma

**Figure 2:**
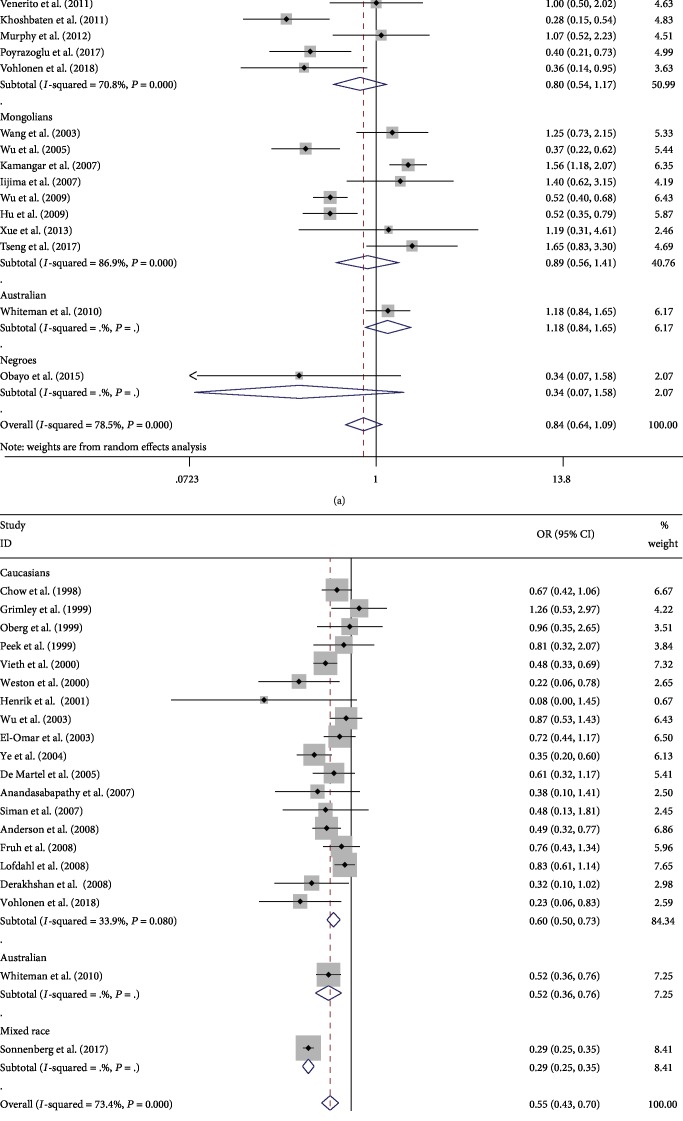
Meta-analysis of the association between Helicobacter pylori infection and esophageal cancer after grouping based on ethnicity: (a) forest plot of esophageal squamous cell carcinoma; (b) forest plot of esophageal adenocarcinoma. Each horizontal bar summarizes a study. Bars represent 95% CIs. Gray squares inform on each of the studies' weight in the meta-analysis. Diamond in the lower part of the graph depicts the pooled estimate along with 95% CIs.

**Figure 3:**
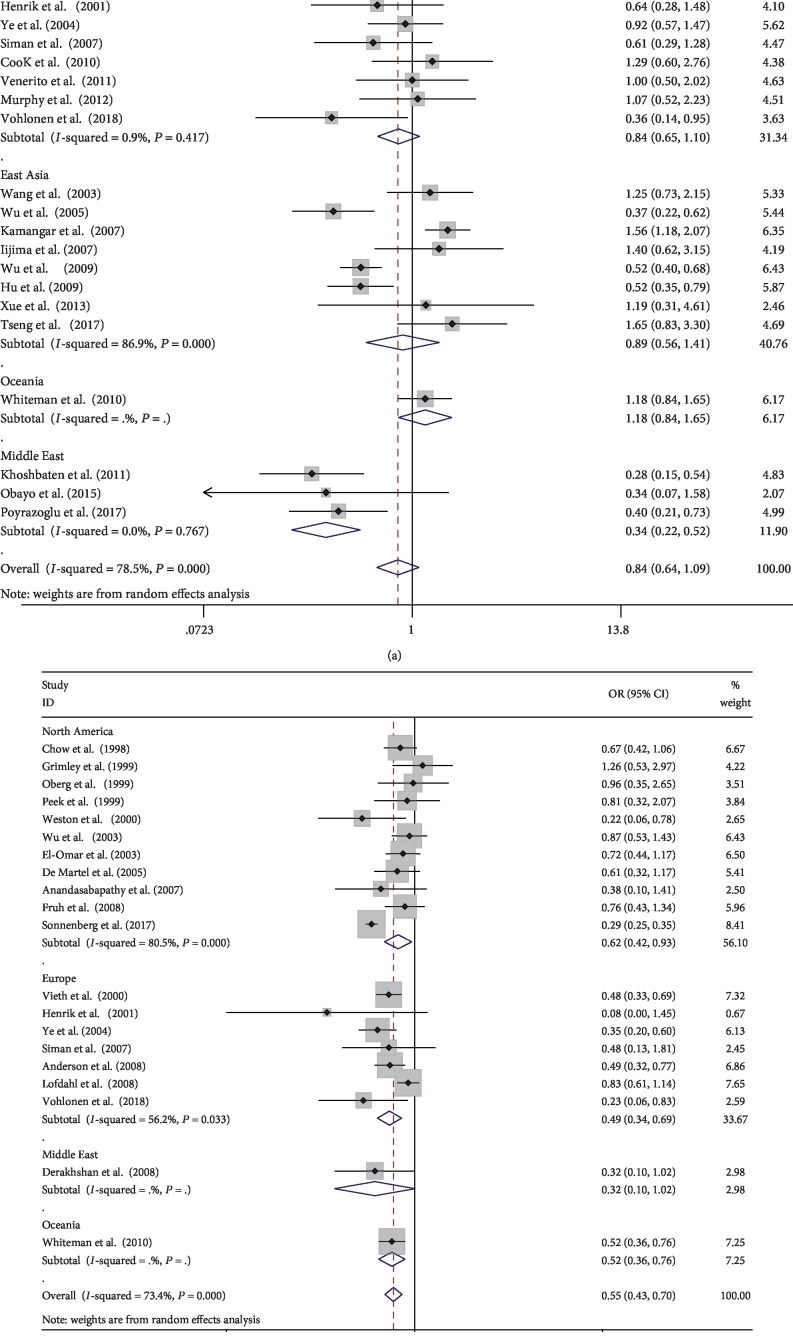
Meta-analysis of the association between Helicobacter pylori infection and esophageal cancer after grouping based on region: (a) forest plot of esophageal squamous cell carcinoma; (b) forest plot of esophageal adenocarcinoma. Each horizontal bar summarizes a study. Bars represent 95% CIs. Gray squares inform on each of the studies' weight in the meta-analysis. Diamond in the lower part of the graph depicts the pooled estimate along with 95% CIs.

Twenty-one studies with 37,114 patients reported the relationship between Helicobacter pylori and esophageal squamous cell carcinoma [17, 20, 26, 27, 29–38, 43–45, 47, 49, 51, 52]. The prevalence of Helicobacter pylori infection in the case group was higher than that in the control group (1,059/2,063 (51.33%) vs. 13,688/35,051 (39.05%)). No statistical significance was showed according to the DerSimonian-Laird method (OR: 0.84, 95% CI: 0.64-1.09). There was no significant association between Helicobacter pylori infection and the risk of esophageal squamous cell carcinoma according to ethnicity, study type, and matching, which had no statistically significant differences between the groups. However, in the Middle East, significantly lower risk of esophageal squamous cell carcinoma was observed after grouping by the study area (OR: 0.34, 95% CI: 0.22-0.52), while that in North America showed the opposite—higher risk (OR: 1.83, 95% CI: 1.17-2.87). Besides, no significant correlation was found in East Asia, Europe, and Australia. Above all, there were significant statistical differences among regions (*P* < 0.001). When analyzing population-based studies, the correlation coefficient between Helicobacter pylori infection and the risk of ESCC was 0.93 (95% CI: 0.68-1.28). Based on clinical studies, with a pooled OR of 0.66 (95% CI: 0.40-1.07). There were significant differences between the groups (*P* = 0.005). After grouping based on the detection method of Helicobacter pylori, the pooled OR of the detection group with various methods was 1.32 (95% CI: 0.87-2.01). There were significant differences between the groups (*P* = 0.003). According to the HKSJ method, Helicobacter pylori did not increase the risk of esophageal squamous cell carcinoma in North America (OR: 1.79, 95% CI: 0.25-12.9). There was no statistically significant difference between the remaining comparative according to the DL methods and the HKSJ method. (The specific results are shown in Table 3(a) and Figures 2(a) and 3(a).)

In the study of esophageal squamous cell carcinoma, the *Q* statistic was significant (*P* < 0.001) and the *I*^2^ statistic had a higher heterogeneity (*I*^2^ = 78.5%). Hence, we performed a subgroup analysis to further explore the potential source of heterogeneity. The heterogeneity did not decrease significantly when grouped according to ethnicity, study object, and matching, but it decreased significantly when grouped according to the study area, study type, and Helicobacter pylori detection method. The results of the sensitivity analysis showed that after excluding one study, the pooling effect of the remaining studies was basically the same as the original total pooling effect. This confirmed that our results were stable.

**Figure 4:**
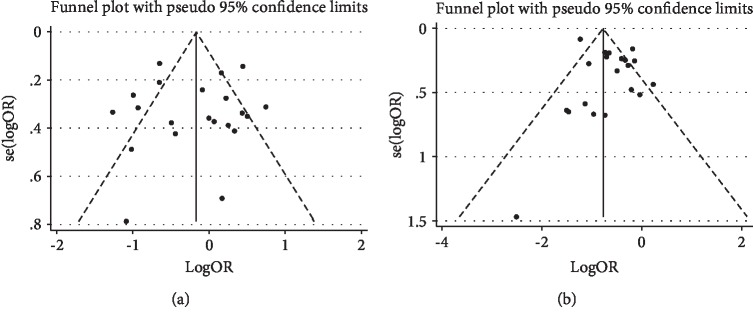
Funnel plot of the association between Helicobacter pylori infection and esophageal cancer: (a) esophageal squamous cell carcinoma; (b) esophageal adenocarcinoma.

The results of the funnel plot showed no significant asymmetry (Figure 4(a)), which suggested that the results were less likely to be affected by publication bias. Harbord's test showed that the *P* values were 0.920, which indicated that there was no significant publication bias in the whole study.

### 3.4. Helicobacter pylori Infection and the Risk of Esophageal Adenocarcinoma

A total of 308,772 patients in 20 studies of esophageal adenocarcinoma were enrolled in our study [18–20, 26, 28, 35, 39–43, 46, 48–50, 52–56]. The prevalence of Helicobacter pylori infection in the case group was not significantly different from that in the control group (682/7,687 (8.87%) vs. 29,109/301,085 (9.67%)). According to the DL method, the quantitative meta-analysis showed that the pooled ORs were 0.55 (95% CI: 0.43-0.70). When grouped according to matching, the OR of the nonmatching group and matching group was 0.48 (95% CI: 0.36-0.65) and 0.73 (95% CI: 0.57-0.92), respectively. There were significant differences between the groups (*P* < 0.001). After grouping based on the detection method of Helicobacter pylori, the OR of the detection group with various methods was 0.81 (95% CI: 0.32-2.07). There were significant differences between the groups (*P* < 0.001). Besides, the risk of Helicobacter pylori infection and esophageal adenocarcinoma was also negatively correlated after grouping based on ethnicity and study object; there were significant differences between the groups (*P* < 0.001). According to the HKSJ method, the pooled OR of the nonmatching group was 0.21 (95% CI: 0.13-0.36), while the OR of the matching group was 0.71 (95% CI: 0.60-0.84). (The specific results are shown in Table 3(b) and Figures 2(b) and 3(b).)

In esophageal adenocarcinoma, the *Q* statistic was significant (*P* < 0.001) and the *I*^2^ statistic had a higher heterogeneity in the study results (*I*^2^ = 73.4%). The heterogeneity did not decrease significantly when grouped according to the study area and study type, but it decreased significantly when grouped according to ethnicity, study object, matching, and detection method of Helicobacter pylori. Besides, we did not find substantial changes in the corresponding pooled ORs by sensitivity analysis. This confirmed that our results were stable.

The results of the funnel plot showed slightly asymmetric signs (Figure 4(b)), which suggested that there may exist potential publication bias. But when we performed the trim and fill method to identify and correct the asymmetry of the funnel plot caused by the potential publication bias, there was no possibility to perform the fill statistics, indicating that no publication bias was detected. Harbord's test showed that the *P* value was 0.222, which indicated that there was no significant publication bias in the whole study.

### 3.5. Metaregression

In order to further study the effects of these characteristics on estimating the relationship between esophageal cancer and Helicobacter pylori, we conducted the metaregression analysis. Year, ethnicity, study area, study type, study object, matching, and Helicobacter pylori detection method were entered as explanatory covariates. First of all, univariate metaregression analysis was performed. Regarding esophageal squamous cell carcinoma, in univariate metaregression analysis, year (*P* = 0.135), ethnicity (Caucasians: *P* = 0.394, Mongolians: *P* = 0.341, and Australian: *P* = 0.279), study area (North America: *P* = 0.462, Europe: *P* = 0.461, East Asia: *P* = 0.533, and Middle East: *P* = 0.039), study type (*P* = 0.510), study object (*P* = 0.226), matching (*P* = 0.959), and detection method (serology: *P* = 0.139; serology, histology, and rapid urease test: *P* = 0.052) were assessed independently. In esophageal adenocarcinoma, year (*P* = 0.022), ethnicity (Caucasians: *P* = 0.009, Australian: *P* = 0.135), study area (North America: *P* = 0.717, Europe: *P* = 0.853, Middle East: *P* = 0.557), study type (*P* = 0.245), study object (*P* = 0.512), matching (*P* = 0.073), and detection method (serology: *P* = 0.626, histology: *P* = 0.207) were also assessed independently. The results of the univariate analysis were presented in Table 4. If the regression coefficient of the covariate was significant at the level of 0.1, then the covariate was entered into the multivariate metaregression. In esophageal squamous cell carcinoma, the study area (North America: *P* = 0.455, Europe: *P* = 0.368, East Asia: *P* = 0.395, Middle East: *P* = 0.05) and detection method (serology: *P* = 0.671; serology, histology, and rapid urease test: *P* = 0.752) were assessed simultaneously. In esophageal adenocarcinoma, year (*P* = 0.49), ethnicity (Caucasians: *P* = 0.361, Australian: *P* = 0.306), and matching (*P* = 0.209) were also assessed simultaneously. The results of the multivariate analysis were presented in Table 5.

## 4. Discussion

This meta-analysis was based on 35 studies with 345,886 patients, which is much larger than the previous data. Our meta-analysis showed no significant correlation between Helicobacter pylori infection and esophageal squamous cell carcinoma in the general population. Some studies have reported similar results [36, 43]. However, some researchers believed that Helicobacter pylori infection may play a protective role in the risk of esophageal squamous cell carcinoma [37, 47]. Another result of our meta-analysis seemed to explain this inconsistency. The risk of esophageal squamous cell carcinoma varies from region to region. Due to the considerable heterogeneity, we also use the HKSJ method for statistics. Compared to the nonsignificant difference in other regions, lower risk of esophageal squamous cell carcinoma was found in the Middle East. It is different from the DL method whose result showed no increase of the risk in North America. But some scholars believed that Helicobacter pylori infection can cause esophageal squamous cell carcinoma through gastric atrophy which may promote the excessive growth of bacteria and increase the production of endogenous nitrosamines, then lead to the esophageal squamous cell carcinoma [49, 57, 58]. However, ESCC is more common in nonindustrialized countries. Smoking and alcohol consumption are the main risk factors which have been proven to be associated with a multiplied risk of the development of esophageal cancer in Western countries [59, 60]. But no significant impact of the two factors were found in northern Iran [61, 62]. The difference of risk factors in different regions may result in the different relationships. Moreover, this difference may also be caused by different dietary cultures in different regions. Multiple detection methods can reduce the false negative of Helicobacter pylori diagnosis. Our results showed that different detection methods were statistically significant that the influence of the false negative cannot be excluded. Besides, some studies pointed that Helicobacter pylori may spontaneously disappear with a progression of gastric atrophy or metaplasia, which leads to false negative and may also have a potential impact on the results [63, 64].

As for esophageal adenocarcinoma, we found that Helicobacter pylori infection may reduce its risk, which is consistent with several previous meta-analyses [4, 14, 15, 65]. At the same time, the Helicobacter pylori infection rate has decreased year by year. There are currently more reliable assumptions about this phenomenon: (1) Helicobacter pylori infection, accompanied by atrophy of gastric body and loss of parietal cells, resulting in reduced reflux, which reduces the incidence of reflux esophagitis and Barrett's esophagus [66]; (2) Helicobacter pylori infection can induce apoptosis of esophageal adenocarcinoma cells progressing from Barrett's esophagus through Fas apoptotic pathway mediated by Caspase [60]. But there are also some other claims that Helicobacter pylori infection is a risk factor for esophageal adenocarcinoma. On the one hand, gastrin induced by Helicobacter pylori is a carcinogenic growth factor, which contributes to the canceration of the esophagus and stomach, especially playing a potential causal role in the progression of Barrett's esophageal neoplasm. On the other hand, Helicobacter pylori induces the expression of nuclear factor-kappa B (NF-*κ*B) and cyclooxygenase- (COX-) 2 in esophageal epithelial cells and plays a role in the inflammation associated with Barrett's esophagus and tumorigenesis in the esophagus [59]. Studies have found that the prevalence of esophageal adenocarcinoma with persistent Helicobacter pylori infection is higher than that after eradication therapy [62]. When matching the control group and the case group, the two different statistical methods showed that the pooled OR of the matching group was significantly higher than that of the nonmatching group. The protective effect of Helicobacter pylori on adenocarcinoma was not so obvious, and there were significant differences between the groups (*P* < 0.001). The incidence of esophageal adenocarcinoma increases with age, and there is male predominance in esophageal adenocarcinoma. The incidence of esophageal adenocarcinoma in men and women is 6 : 1 in general, which is as high as 8 : 1 in some of the other populations, such as in the United States [61, 67]. Therefore, the matching of the case group and the control group may make a great difference in the results. Our meta-analysis showed that the prevalence of Helicobacter pylori was almost the same between the case group and the control group. Take et al. [68] conducted a 20-year study of 2,782 patients and concluded that the risk of esophageal adenocarcinoma caused by Helicobacter pylori eradication may be unfounded. Besides, epidemiological studies have shown that the incidence of gastroesophageal reflux disease, Barrett's esophagus, and distal esophageal cancer is lower in Malaysians with lower prevalence of Helicobacter pylori infection [69]. Moreover, we grouped the detection methods of Helicobacter pylori and found that the pooled OR of multiple detection methods was 0.81 (95% CI: 0.32-2.07). A variety of detection methods can minimize false negative as far as possible. Due to the limited number of the articles, further research needs to be performed in the future. Although current evidence suggests that Helicobacter pylori infection may reduce the risk of esophageal adenocarcinoma, this claim may be one-sided, and the “protective effect” of Helicobacter pylori infection may be overestimated. We found that there were significant statistical differences among ethnicities. This might be because the etiology of esophageal adenocarcinoma is related to the genetic factors and is attributed to the mutations in the lineage [70].

Heterogeneity might affect the interpretation of the results. This meta-analysis showed that there was considerable heterogeneity. Regarding esophageal squamous cell carcinoma, we performed a subgroup analysis of six possible factors. It was found that when grouped according to the study area, study type, and detection of Helicobacter pylori, heterogeneity decreased significantly. We then conducted metaregression and found that the study area may contribute more to the overall heterogeneity. As we have found, the risk of esophageal squamous cell carcinoma varies from area to area. In esophageal adenocarcinoma, subgroup analysis demonstrated that the heterogeneity was significantly lower after grouping by ethnicity, study object, matching, and detection of Helicobacter pylori. Metaregression showed that matching might contribute little to the overall heterogeneity; then, year and ethnicity may contribute more to the overall heterogeneity. However, multivariate regression analysis showed that year and ethnicity did not significantly differ. This may be related to the annual concentration of the articles we have included based on ethnicity.

The advantages of our meta-analysis are as follows: Firstly, compared to the previous meta-analyses, our study included cohort studies, and the number of enrolled studies was more reliable. Subgroup analysis and metaregression were conducted to identify potential sources of heterogeneity; then, this study analyzed whether the differences between the groups were statistically significant. Secondly, all the studies in our meta-analysis have acceptable quality, the results of sensitivity analysis are stable, and the impact of publication bias is small. Thirdly, we analyzed the relationship between Helicobacter pylori infection and the risk of esophageal cancer in more details, whether different ethnicities and regions are the same and whether the differences have statistical significance. However, this study still has limitations. Firstly, this meta-analysis has not been registered online, which may cause bias in the analysis. Secondly, a special disadvantage of most original studies is the confounding factor; that is, many factors are not taken into account in research design or data analysis. Moreover, current epidemiological studies provide uncertain data on the negative or neutral correlation between Helicobacter pylori infection and esophageal adenocarcinoma. The analysis of observational studies supports the negative correlation that we have not analyzed the differences. Finally, the heterogeneity of the total combined effects of esophageal squamous cell carcinoma and esophageal adenocarcinoma is high in our study. Although these heterogeneities can be reduced after subgroup analysis, they might lead to a reduction in the number of studies and thus limit the reliability of data.

## 5. Conclusions

In summary, no significant association was found between Helicobacter pylori infection and the risk of esophageal squamous cell carcinoma in the general population. However, lower risk was found in the Middle East when grouped by the study area. In the ethnic stratification analysis, there was no significant correlation between Helicobacter pylori infection and the risk of esophageal squamous cell carcinoma. Helicobacter pylori infection may reduce the risk of esophageal adenocarcinoma in the general population, but this may be one-sided; the statement of “protection effect” may be overestimated. Therefore, well-designed prospective cohort studies with a powered sample size are required, in which potential confounders should be taken into account to validate their relationship.

## Figures and Tables

**Table 1:** Characteristics of literatures included in the meta-analysis.

First author	Study country	Ethnicity	Study type	Study object	Age^∗^	Sex^§^ (% male)	Matched^∗∗^	Follow-up
Talley et al.	America	Caucasians	Case-control	Clin (S)	63	49	Yes	—
Chow et al.	America	Caucasians	Case-control	Pop (A)	—	—	Yes	—
Grimley et al.	America	Caucasians	Case-control	Clin (A)	70	70-78	No	—
Öberg et al.	America	Caucasians	Case-control	Clin (A)	—	—	No	—
Peek et al.	America	Caucasians	Case-control	Clin (A)	61	97	No	—
Vieth et al.	Germany	Caucasians	Case-control	Clin (A)	—	—	—	—
Weston et al.	America	Caucasians	Case-control	Clin (A)	60.6	100	No	—
Henrik et al.	Sweden	Caucasians	Case-control	Pop (S, A)	49.6	—	No	—
Wu et al.	America	Caucasians	Case-control	Pop (A)	—	—	Yes	—
El-Omar et al.	America	Caucasians	Case-control	Pop (S, A)	S: 66 A: 65	S: 89 A: 86	S: No A: Yes	—
Wang et al.	China	Mongolians	Case-control	Pop (S)	—	—	No	—
Ye et al.	Sweden	Caucasians	Case-control	Pop (S, A)	S: 64 A: 69	S: 69 A: 91	No No	—
De Martel et al.	America	Caucasians	Case-control	Pop (A)	47.9	80.4	Yes	—
Wu et al.	China	Mongolians	Case-control	Pop (S)	—	91.3	No	—
Anandasabapathy et al.	America	Caucasians	Case-control	Clin (A)	59.23	94.3	No	—
Kamangar et al.	China	Mongolians	Case-control	Pop (S)	54.5	46.3	No	—
Iijima et al.	Japan	Mongolians	Case-control	Clin (S)	68.6	90.4	No	—
Simán et al.	Sweden	Caucasians	Case-control	Pop (S, A)	S: 50.6 A: 49.3	S: 81.1 A: 91.7	No No	—
Anderson et al.	Ireland	Caucasians	Case-control	Pop (A)	64.2	84	No	—
Früh et al.	Canada	Caucasians	Case-control	Clin (A)	64	88	Yes	—
Löfdahl et al.	Sweden	Caucasians	Case-control	Pop (A)	—	—	No	—
Derakhshan et al.	Iran	Caucasians	Case-control	Clin (A)	63.9	63.2	No	—
Wu et al.	China	Mongolians	Case-control	Pop (S)	58.3	95	—	—
Hu et al.	China	Mongolians	Case-control	Clin (S)	—	97	Yes	—
Whiteman et al.	Australia	Australian	Case-control	Pop (S, A)	—	S: 58 A: 92	No No	—
Cook et al.	Finland	Caucasians	Case-control	Pop (S)	57.7	—	No	—
Venerito et al.	Germany	Caucasians	Case-control	Clin (S)	64.9	69.3	No	—
Khoshbaten et al.	Iran	Caucasians	Case-control	Clin (S)	63.9	64	No	—
Murphy et al.	Finland	Caucasians	Case-control	Pop (S)	57.9	—	No	—
Xue et al.	China	Mongolians	Cohort	Pop (S)	45.29	36.91	—	15 y
Obayo et al.	Uganda	Negroes	Case-control	Clin (S)	—	—	—	—
Poyrazoglu et al.	Iran	Caucasians	Case-control	Clin (S)	—	—	No	—
Sonnenberg et al.	America	Mixed race	Case-control	Clin (A)	66.8	79.3	No	—
Tseng et al.	China	Mongolians	Case-control	Pop (S)	—	—	—	—
Vohlonen et al.	Finland	Caucasians	Cohort	Pop (S, A)	—	100	No	15 y

Note: ^∗^average or median age in case group; ^§^the proportion of males in the case group; ^∗∗^does the case group match the age, sex, or race of the control group? Clin: clinical-based; Pop: population-based; S: esophageal squamous cell carcinoma; A: esophageal adenocarcinoma.

**Table:** (a) The data of Helicobacter pylori infection in esophageal squamous cell carcinoma

First author	Year	Esophageal squamous cell carcinoma				Hp
		Case		Control		
		Hp+	Hp-	Hp+	Hp-	Test method
Talley et al.	1991	20	21	96	156	S
Henrik et al.	2001	10	19	67	82	S
El-Omar et al.	2003	31	22	84	126	S
Wang et al.	2003	33	30	145	165	S
Ye et al.	2004	32	53	198	301	S
Wu et al.	2005	28	99	74	97	S
Kamangar et al.	2007	254	81	662	330	S
Iijima et al.	2007	60	13	56	17	S, H, U
Simán et al.	2007	15	22	68	61	S
Wu et al.	2009	112	205	563	540	S
Hu et al.	2009	66	114	102	92	S
Whiteman et al.	2010	54	154	302	1,014	S
Cook et al.	2010	64	14	71	20	S
Venerito et al.	2011	53	22	53	22	S, H, U
Khoshbaten et al.	2011	58	42	83	17	S
Murphy et al.	2012	64	18	63	19	S
Xue et al.	2013	7	3	988	503	S
Obayo et al.	2015	14	3	69	5	U
Poyrazoglu et al.	2017	66	30	128	23	U
Tseng et al.	2017	12	25	3,638	12,541	S, H, U
Vohlonen et al.	2018	6	14	6,178	5,232	S

Note: Hp: Helicobacter pylori; S: serology; H: histology; U: rapid urease test.

**Table:** (b) The data of Helicobacter pylori infection in esophageal adenocarcinoma

First author	Year	Esophageal adenocarcinoma				Hp
		Case		Control		
		Hp+	Hp-	Hp+	Hp-	Test method
Chow et al.	1998	38	91	86	137	S
Grimley et al.	1999	24	16	25	21	S
Öberg et al.	1999	5	32	32	197	H
Peek et al.	1999	11	19	20	28	S, H
Vieth et al.	2000	66	72	468	244	H
Weston et al.	2000	3	17	96	121	H
Henrik et al.	2001	0	7	67	82	S
Wu et al.	2003	49	31	230	126	S
El-Omar et al.	2003	35	73	84	126	S
Ye et al.	2004	18	79	198	301	S
De Martel et al.	2005	19	32	74	76	S
Anandasabapathy et al.	2007	4	21	10	20	H
Simán et al.	2007	4	8	24	23	S
Anderson et al.	2008	55	68	157	96	S
Früh et al.	2008	36	64	43	58	S
Löfdahl et al.	2008	130	100	304	195	S
Derakhshan et al.	2008	9	10	28	10	S
Whiteman et al.	2010	35	225	302	1,014	S
Sonnenberg et al.	2017	13	6,029	20,683	263,869	H
Vohlonen et al.	2018	3	11	6,178	5,232	S

Note: Hp: Helicobacter pylori; S: serology; H: histology; U: rapid urease test.

**Table:** (a) Subgroup comparisons for Helicobacter pylori infection on the risk of esophageal squamous cell carcinoma

Subgroup	No. of studies	*I* ^2^ (%)	Overall OR (95% CI)		*P* value^§^
			DL	HKSJ	
Esophageal squamous cell carcinoma					
Case/control (2,063/35,051)					
All studies	21	78.5	0.84 (0.64, 1.09)	0.74 (0.54, 0.97)	
Ethnicity					0.144
Caucasians	11	70.8	0.80 (0.54, 1.17)	0.73 (0.48, 1.11)	
Mongolians	8	86.9	0.89 (0.56, 1.41)	1.13 (0.79, 1.60)	
Negroes	1	—	0.34 (0.07, 1.58)	—	
Australian	1	—	1.18 (0.84, 1.65)	—	
Study area					<0.001^∗^
North America	2	0	1.83 (1.17, 2.87)	1.79 (0.25, 12.9)	
East Asia	8	86.9	0.89 (0.56, 1.41)	1.13 (0.79, 1.60)	
Europe	7	0.9	0.84 (0.65, 1.10)	0.72 (0.46, 1.12)	
Middle East	3	0	0.34 (0.22, 0.52)	0.34 (0.26, 0.44)	
Oceania	1	—	1.18 (0.84, 1.65)	—	
Study type					0.250
Case-control	19	80	0.85 (0.65, 1.13)	0.70 (0.51, 0.97)	
Cohort	2	49	0.59 (0.19, 1.86)	0.80 (0.0, 967.18)	
Study object					0.005^∗^
Population-based studies	14	79.5	0.93 (0.68, 1.28)	0.91 (0.66, 1.24)	
Clinical-based studies	7	72.5	0.66 (0.40, 1.07)	0.53 (0.28, 0.99)	
Matching					0.290
Studies with matched controls	2	86.5	0.87 (0.30, 2.52)	1.14 (0.0, 563.04)	
Studies without matched controls	19	78.7	0.83 (0.63, 1.11)	0.71 (0.52, 0.96)	
Helicobacter pylori detection method					0.003^∗^
Serology	16	81.4	0.83 (0.61, 1.12)	0.83 (0.61, 1.14)	
Rapid urease test	2	0	0.39 (0.22, 0.69)	0.35 (0.17, 0.69)	
Serology, histology, and rapid urease test	3	0	1.32 (0.87, 2.01)	1.33 (0.72, 2.44)	

Note: DL: DerSimonian-Laird; HKSJ: Hartung-Knapp-Sidik-Jonkman. ^§^Differences between subgroups; ^∗^the difference was statistically significant.

**Table:** (b) Subgroup comparisons for Helicobacter pylori infection on the risk of esophageal adenocarcinoma

Subgroup	No. of studies	*I* ^2^ (%)	Overall OR (95% CI)		*P* value^§^
			DL	HKSJ	
Esophageal adenocarcinoma					
Case/control (7687/301085)					
All studies	20	73.4	0.55 (0.43, 0.70)	0.23 (0.15, 0.36)	
Ethnicity					<0.001^∗^
Caucasians	18	33.9	0.60 (0.50, 0.73)	0.23 (0.14, 0.37)	
Mixed race	1	—	0.29 (0.25, 0.35)	—	
Australian	1	—	0.52 (0.36, 0.76)	—	
Study area					0.100
North America	11	80.5	0.62 (0.42, 0.93)	0.54 (0.35, 0.81)	
Europe	7	56.2	0.49 (0.34, 0.69)	0.13 (0.06, 0.27)	
Middle East	1	—	0.32 (0.10, 1.02)	—	
Oceania	1	—	0.52 (0.36, 0.76)	—	
Study type					0.287
Case-control	19	74.4	0.56 (0.44, 0.72)	0.23 (0.14, 0.37)	
Cohort	1	—	0.23 (0.06, 0.83)	—	
Study object					<0.001^∗^
Population-based studies	11	37.9	0.59 (0.48, 0.73)	0.15 (0.08, 0.28)	
Clinical-based studies	9	73.4	0.52 (0.35, 0.79)	0.47 (0.29, 0.77)	
Matching					<0.001^∗^
Studies with matched controls	5	0	0.73 (0.57, 0.92)	0.71 (0.60, 0.84)	
Studies without matched controls	15	73.3	0.48 (0.36, 0.65)	0.21 (0.13, 0.36)	
Helicobacter pylori detection method					<0.001^∗^
Serology	14	36.3	0.62 (0.51, 0.75)	0.19 (0.11, 0.32)	
Histology	5	62.1	0.39 (0.26, 0.60)	0.39 (0.18, 0.84)	
Serology, histology	1	—	0.81 (0.32, 2.07)	—	

Note: DL: DerSimonian-Laird; HKSJ: Hartung-Knapp-Sidik-Jonkman. ^§^Differences between subgroups; ^∗^the difference was statistically significant.

**Table 4:** Univariate metaregression analysis for the potential variables between studies.

Covariates	Coefficient	Standard error	*t*	*P*	95% confidence interval	
ESCC						
Year	-0.034	0.022	-1.56	0.135	-0.080	0.011
Ethnicity						
Caucasians	0.855	0.977	0.88	0.394	-1.206	2.917
Mongolians	0.961	0.982	0.98	0.341	-1.111	3.033
Australian	1.247	1.114	1.12	0.279	-1.103	3.598
Study area						
North America	0.434	0.577	0.75	0.462	-0.788	1.656
Europe	-0.370	0.490	-0.76	0.461	-1.408	0.669
East Asia	-0.304	0.477	-0.64	0.533	-1.315	0.707
Middle East	-1.254	0.559	-2.24	0.039^∗^	-2.439	-0.692
Study type	0.379	0.564	0.67	0.510	-0.803	1.560
Study object	0.356	0.284	1.25	0.226	-0.239	0.950
Matching	0.024	0.447	0.05	0.959	-0.911	0.959
Detection method						
S	0.785	0.508	1.55	0.139	-0.281	1.852
S, H, U	1.251	0.600	2.08	0.052	-0.010	2.512
EAC						
Year	-0.040	0.016	-2.50	0.022^∗^	-0.073	-0.006
Ethnicity						
Caucasians	0.729	0.249	2.93	0.009^∗^	0.204	1.254
Australian	0.581	0.370	1.57	0.135	-0.199	1.362
Study area						
North America	0.163	0.442	0.37	0.717	-0.774	1.100
Europe	-0.866	0.459	-0.19	0.853	-1.059	0.885
Middle East	-0.485	0.808	-0.60	0.557	-2.199	1.228
Study type	0.883	0.735	1.20	0.245	-0.661	2.426
Study object	-0.142	0.212	-0.67	0.512	-0.587	0.304
Matching	0.404	0.212	1.90	0.073	-0.421	0.849
Detection method						
S	-0.278	0.560	-0.50	0.626	-1.461	0.903
H	-0.757	0.577	-1.31	0.207	-1.974	0.460

Note: ESCC: esophageal squamous cell carcinoma; EAC: esophageal adenocarcinoma; S: serology; H: histology; U: rapid urease test. ^∗^the difference was statistically significant.

**Table 5:** Multivariate metaregression analysis for the potential variables between studies.

Covariates	Coefficient	Standard error	*t*	*P*	95% confidence interval	
ESCC						
Study area						
North America	0.434	0.566	0.77	0.455	-0.779	1.647
Europe	0.449	0.483	-0.93	0.368	-1.485	0.586
East Asia	-0.414	0.472	-0.88	0.395	-1.427	0.599
Middle East	-1.426	0.677	-2.11	0.05^∗^	-2.878	0.026
Detection method						
S	-0.296	0.681	-0.43	0.671	-1.757	1.165
S, H, U	0.246	0.765	0.32	0.752	-1.395	1.888
EAC						
Year	-0.017	0.024	-0.71	0.490	-0.069	-0.035
Ethnicity						
Caucasians	0.394	0.417	0.94	0.361	-0.496	1.283
Australian	0.461	0.435	1.06	0.306	-0.467	1.389
Matching	0.275	0.209	1.31	0.209	-0.171	0.721

Note: ESCC: esophageal squamous cell carcinoma; EAC: esophageal adenocarcinoma; S: serology; H: histology; U: rapid urease test. ^∗^the difference was statistically significant.

## Appendix

The following are the PubMed Search Terms: (“Esophageal Neoplasm” [MeSH] or “Neoplasm, Esophageal” [MeSH] or “Esophagus Neoplasm” [MeSH] or “Esophagus Neoplasms” [MeSH] or “Neoplasm, Esophagus” [MeSH] or “Neoplasms, Esophagus” [MeSH] or “Neoplasms, Esophageal” [MeSH] or “Esophageal Neoplasms” [MeSH] or “Cancer of Esophagus” [MeSH] or “Cancer of the Esophagus” [MeSH] or “Esophagus Cancer” [MeSH] or “Cancer, Esophagus” [MeSH] or “Cancers, Esophagus” [MeSH] or “Esophagus Cancers” [MeSH] or “Esophageal Cancer” [MeSH] or “Cancer, Esophageal” [MeSH] or “Cancers, Esophageal” [MeSH] or “Esophageal Cancers” [MeSH] or “esophageal carcinoma” [MeSH] or “esophageal carcinomas” [MeSH] or “Esophagus carcinoma” [MeSH] or “Esophagus carcinomas” [MeSH] or “esophageal tumor” [MeSH] or “esophageal tumors”[MeSH] or “Esophagus tumors” [MeSH] or “Esophagus tumor” [MeSH] or “esophageal squamous cell carcinoma” [MeSH] or “ESCC” [MeSH] or “EAC” [All Fields] or “esophageal squamous carcinoma” [MeSH] or “esophageal adenocarcinoma” [MeSH] or “adenocarcinoma of the esophagus” [MeSH]) and (“Helicobacter pylori” [MeSH] or “H pylori” [MeSH] or “H. pylori” [MeSH] or “HP” [All Fields] or “Helicobacter” [MeSH]).

The following are the Embase Search Terms: (“Esophageal Neoplasm” [Emtree] or “Neoplasm, Esophageal” [Emtree] or “Esophagus Neoplasm” [Emtree] or “Esophagus Neoplasms” [Emtree] or “Neoplasm, Esophagus” [Emtree] or “Neoplasms, Esophagus” [Emtree] or “Neoplasms, Esophageal” [Emtree] or “Esophageal Neoplasms” [Emtree] or “Cancer of Esophagus” [Emtree] or “Cancer of the Esophagus” [Emtree] or “Esophagus Cancer” [Emtree] or “Cancer, Esophagus” [Emtree] or “Cancers, Esophagus” [Emtree] or “Esophagus Cancers” [Emtree] or “Esophageal Cancer” [Emtree] or “Cancer, Esophageal” [Emtree] or “Cancers, Esophageal” [Emtree] or “Esophageal Cancers” [Emtree] or “esophageal carcinoma” [Emtree] or “esophageal carcinomas” [Emtree] or “Esophagus carcinoma” [Emtree] or “Esophagus carcinomas” [Emtree] or “esophageal tumor” [Emtree] or “esophageal tumors” [Emtree] or “Esophagus tumors” [Emtree] or “Esophagus tumor” [Emtree] or “esophageal squamous cell carcinoma” [Emtree] or “ESCC” [Emtree] or “EAC” [Emtree] or “esophageal squamous carcinoma” [Emtree] or “esophageal adenocarcinoma” [Emtree] or “adenocarcinoma of the esophagus” [Emtree]) and (“Helicobacter pylori” [Emtree] or “H pylori” [Emtree] or “H. pylori” [Emtree] or “HP” [Emtree] or “Helicobacter” [Emtree]).

## Abbreviations

ESCC: Esophageal squamous cell carcinoma
EAC: Esophageal adenocarcinoma
HP/H. pylori: Helicobacter pylori
MOOSE: Meta-analysis of Observational Studies in Epidemiology
NOS: The Newcastle-Ottawa Scale
OR: Odds ratio
RR: Relative risk
CI: Confidence interval
NF-*κ*B: Nuclear factor-kappa B
COX-2: Cyclooxygenase-2
DL: DerSimonian-Laird
HKSJ: Hartung-Knapp-Sidik-Jonkman.

## Supplementary Materials

Supplementary Materials Table S1: critical appraisal of the included studies according to the Newcastle-Ottawa Scale. Table S2: assessment of risk of bias in individual studies. Table S3: checklist for Meta-analysis of Observational Studies in Epidemiology (MOOSE).
