# Supplementary material for: Systematic Review with Meta-analysis: Association of Helicobacter pylori Infection with Esophageal Cancer
Source: Gastroenterol Res Pract. 2019 Dec 1;2019:1953497. doi: 10.1155/2019/1953497 (PMC6913313; doi:10.1155/2019/1953497)
Supplement: Supplementary Materials — Table S1: critical appraisal of the included studies according to the Newcastle-Ottawa Scale. Table S2: assessment of risk of bias in individual studies. Table S3: checklist for Meta-analysis of Observational Studies in Epidemiology (MOOSE). [file 1953497.f1.doc]

**Table S1. Critical Appraisal of the Included Studies According to the Newcastle-Ottawa Scale**

| Selection Comparability Exposure/Outcome  Study A B C D E F G H Total | | | | | | | | | |
| --- | --- | --- | --- | --- | --- | --- | --- | --- | --- |
| Talley et al | 1 | 1 | 1 | 1 | 2 | 1 | 1 | 0 | 8 |
| Chow et al | 0 | 1 | 1 | 1 | 2 | 1 | 1 | 1 | 8 |
| Grimley et al | 1 | 1 | 0 | 1 | 1 | 1 | 1 | 1 | 7 |
| Öberg et al | 1 | 1 | 0 | 1 | 1 | 1 | 1 | 1 | 7 |
| Peek et al | 1 | 1 | 0 | 1 | 1 | 1 | 1 | 1 | 7 |
| Vieth et al | 1 | 1 | 0 | 1 | 0 | 1 | 1 | 1 | 6 |
| Weston et al | 1 | 1 | 0 | 1 | 2 | 1 | 1 | 1 | 8 |
| Henrik et al | 0 | 1 | 1 | 1 | 2 | 1 | 1 | 0 | 7 |
| Wu et al | 0 | 1 | 1 | 1 | 2 | 1 | 1 | 1 | 8 |
| El-Omar et al | 1 | 1 | 1 | 1 | 2 | 1 | 1 | 0 | 8 |
| Wang et al | 0 | 1 | 1 | 1 | 2 | 1 | 1 | 0 | 7 |
| Ye et al | 1 | 1 | 1 | 1 | 2 | 1 | 1 | 0 | 8 |
| De Martel et al | 1 | 1 | 1 | 1 | 2 | 1 | 1 | 1 | 9 |
| Wu et al | 1 | 0 | 1 | 1 | 2 | 1 | 1 | 0 | 7 |
| Anandasabapathy et al | 1 | 1 | 0 | 1 | 2 | 1 | 1 | 0 | 7 |
| Kamangar et al | 1 | 1 | 1 | 1 | 2 | 1 | 1 | 0 | 8 |
| Iijima et al | 1 | 0 | 0 | 1 | 2 | 1 | 1 | 0 | 6 |
| Simán et al | 1 | 1 | 1 | 1 | 2 | 1 | 1 | 0 | 8 |
| Anderson et al | 1 | 0 | 1 | 1 | 2 | 1 | 1 | 0 | 7 |
| Früh et al | 1 | 1 | 1 | 1 | 2 | 1 | 1 | 1 | 9 |
| Löfdahl et al | 1 | 1 | 1 | 1 | 2 | 1 | 1 | 0 | 8 |
| Derakhshan et al | 1 | 0 | 0 | 1 | 2 | 1 | 1 | 0 | 6 |
| Wu et al | 1 | 1 | 1 | 1 | 0 | 1 | 1 | 1 | 7 |
| Hu et al | 1 | 0 | 1 | 1 | 2 | 1 | 1 | 0 | 7 |
| Whiteman et al | 1 | 1 | 1 | 1 | 1 | 1 | 1 | 1 | 8 |
| CooK et al | 0 | 1 | 1 | 1 | 1 | 1 | 1 | 1 | 7 |
| Venerito et al | 1 | 1 | 0 | 1 | 2 | 1 | 1 | 1 | 8 |
| Khoshbaten et al | 0 | 1 | 0 | 1 | 2 | 1 | 1 | 1 | 7 |
| Murphy et al | 0 | 1 | 1 | 1 | 2 | 1 | 1 | 1 | 8 |
| Xue et al | 1 | 1 | 1 | 1 | 0 | 1 | 1 | 1 | 7 |
| Obayo et al | 1 | 1 | 0 | 1 | 0 | 1 | 1 | 0 | 5 |
| Poyrazoglu et al | 1 | 1 | 1 | 1 | 2 | 1 | 1 | 1 | 9 |
| Sonnenberg et al | 0 | 1 | 1 | 1 | 1 | 1 | 1 | 1 | 7 |
| Tseng et al | 0 | 1 | 1 | 1 | 0 | 1 | 1 | 0 | 5 |
| Vohlonen et al | 1 | 1 | 1 | 1 | 2 | 1 | 1 | 1 | 9 |

NOTE：A：Is the case definition adequate?/Representativeness of the exposed cohort; B: Representativeness of the cases/Selection of the non-exposed cohort; C: Selection of controls/ Ascertainment of exposure; D: Definition of controls/Demonstration that outcome of interest was not present at start of study; E: Comparability of cases and controls on the basis of the design or analysis/ Comparability of cohorts on the basis of the design or analysis; F: Ascertainment of exposure/ Assessment of outcome; G: Same method of ascertainment for cases and controls/ Was follow-up Long enough for outcomes to occur; H: Non-response rate/ Adequacy of follow up of cohorts.

**Table S2. Assessment of risk of bias in individual studies**

Confounding Selection Bias of Implementation Bias due Measurement Reporting Total

Study bias bias intervention bias to missing bias bias

classification data

| Talley et al | Moderate | Low | Moderate | Moderate | Moderate | Low | Low | Moderate |
| --- | --- | --- | --- | --- | --- | --- | --- | --- |
| Chow et al | Moderate | Low | Low | Moderate | Moderate | Low | Low | Moderate |
| Grimley et al | Moderate | Low | Low | Moderate | Moderate | Low | Low | Moderate |
| Öberg et al | Moderate | Low | Low | Low | Low | Low | Moderate | Moderate |
| Peek et al | Low | Low | Low | Moderate | Moderate | Low | Low | Moderate |
| Vieth et al | Moderate | Moderate | Moderate | Moderate | Low | Low | Low | Moderate |
| Weston et al | Serious | Moderate | Moderate | Moderate | Low | Low | Low | Serious |
| Henrik et al | Low | Low | Low | Low | Low | Low | Low | Low |
| Wu et al | Moderate | Low | Moderate | Moderate | Low | Low | Low | Moderate |
| El-Omar et al | Moderate | Low | Moderate | Moderate | Low | Low | Low | Moderate |
| Wang et al | Moderate | Moderate | Moderate | Moderate | Low | Low | Low | Moderate |
| Ye et al | Moderate | Low | Moderate | Moderate | Low | Low | Low | Moderate |
| De Martel et al | Low | Low | Moderate | Moderate | Low | Low | Low | Moderate |
| Wu et al | Moderate | Low | Moderate | Low | Low | Low | Low | Moderate |
| Anandasabapathy et al | Serious | Low | Moderate | Moderate | Low | Low | Low | Serious |
| Kamangar et al | Low | Low | Low | Low | Low | Low | Low | Low |
| Iijima et al | Moderate | Low | Moderate | Moderate | Low | Low | Low | Moderate |
| Simán et al | Moderate | Low | Moderate | Moderate | Low | Low | Low | Moderate |
| Anderson et al | Moderate | Low | Moderate | Moderate | Moderate | Low | Low | Moderate |
| Früh et al | Moderate | Low | Moderate | Moderate | Low | Low | Low | Moderate |
| Löfdahl et al | Moderate | Low | Low | Moderate | Low | Low | Low | Moderate |
| Derakhshan et al | Low | Moderate | Moderate | Moderate | Low | Low | Low | Moderate |
| Wu et al | Serious | Low | Moderate | Moderate | Low | Low | Low | Serious |
| Hu et al | Moderate | Low | Moderate | Moderate | Low | Low | Low | Moderate |
| Whiteman et al | Moderate | Low | Moderate | Moderate | Low | Low | Low | Moderate |
| CooK et al | Low | Low | Low | Low | Low | Low | Low | Low |
| Venerito et al | Moderate | Low | Moderate | Moderate | Moderate | Low | Low | Moderate |
| Khoshbaten et al | Serious | Moderate | Moderate | Moderate | Moderate | Low | Low | Serious |
| Murphy et al | Low | Low | Low | Low | Low | Low | Low | Low |
| Xue et al | Moderate | Low | Moderate | Moderate | Moderate | Low | Low | Moderate |
| Obayo et al | Moderate | Low | Moderate | Moderate | Moderate | Low | Low | Moderate |
| Poyrazoglu et al | Moderate | Low | Moderate | Moderate | Low | Low | Low | Moderate |
| Sonnenberg et al | Low | Low | Low | Low | Low | Low | Low | Low |
| Tseng et al | Moderate | Low | Moderate | Moderate | Moderate | Low | Low | Moderate |
| Vohlonen et al | Moderate | Moderate | Moderate | Moderate | Moderate | Low | Low | Moderate |

**Table S3. Checklist for Meta-analysis of Observational Studies in Epidemiology (MOOSE)**

| Item no. Recommendation Reported on page no. | |
| --- | --- |
| Reporting of background includes  1 Problem deﬁnition | P4 |
| 2 Hypothesis statement | P4 |
| 3 Description of study outcome(s) | P4 |
| 4 Type of exposure or intervention used | P3-4 |
| 5 Type of study designs used | P3-4 |
| 6 Study population | P4 |
| Reporting of search strategy includes |  |
| 7 Qualiﬁcations of searchers (eg, librarians and investigators) | P5 |
| 8 Search strategy, including time period included in the synthesis and key words  9 Effort to include all available studies, including contact with authors  10 Databases and registries searched  11 Search software used, name and version, including special features used  (eg, explosion)  12 Use of hand searching (eg, reference lists of obtained articles )  13 List of citations located and those excluded, including justiﬁcation  14 Method of addressing articles published in languages other than English  15 Method of handling abstracts and unpublished studies  16 Description of any contact with authors  Reporting of methods includes  17 Description of relevance or appropriateness of studies assembled for assessing the  hypothesis to be tested  18 Rationale for the selection and coding of data (eg, sound clinical principles  or convenience)  19 Documentation of how data were classiﬁed and coded (eg, multiple raters,  blinding and interrater reliability )  20 Assessment of confounding (eg, comparability of cases and controls in studies where  appropriate)  21 Assessment of study quality, including blinding of quality assessors, stratiﬁcation or  regression on possible predictors of study results  22 Assessment of heterogeneity  23 Description of statistical methods (eg, complete description of ﬁxed or random effects  models, justiﬁcation of whether the chosen models account for predictors of study  results, dose-response models, or cumulative meta-analysis) in sufﬁcient detail to  be replicated  24 Provision of appropriate tables and graphics  Reporting of results includes  25 Graphic summarizing individual study estimates and overall estimate  26 Table giving descriptive information for each study included  27 Results of sensitivity testing (eg, subgroup analysis)  28 Indication of statistical uncertainty of ﬁnding | P5, Appendix 1  P5  P5  P5    P5  P5  -  P5  -    P5    -    -  P6  P6-7    P7  P7-8      Table1,2,S1,S2;Figure4  Table3;Figure2,3  Table1,2  Table3,4,5  - |

**Appendix 1**

PubMed search terms:

(“Esophageal Neoplasm”[MeSH] or “Neoplasm, Esophageal”[MeSH]or “Esophagus Neoplasm” [MeSH] or “Esophagus Neoplasms” [MeSH] or “Neoplasm, Esophagus” [MeSH] or “Neoplasms, Esophagus” [MeSH] or “Neoplasms, Esophageal” [MeSH] or “Esophageal Neoplasms” [MeSH] or “Cancer of Esophagus” [MeSH] or “Cancer of the Esophagus” [MeSH] or “Esophagus Cancer” [MeSH] or “Cancer, Esophagus” [MeSH] or “Cancers, Esophagus” [MeSH] or “Esophagus Cancers” [MeSH] or “Esophageal Cancer” [MeSH] or “Cancer, Esophageal” [MeSH] or “Cancers, Esophageal” [MeSH] or “Esophageal Cancers” [MeSH] or “esophageal carcinoma” [MeSH] or “esophageal carcinomas” [MeSH] or “Esophagus carcinoma” [MeSH] or “Esophagus carcinomas” [MeSH] or “esophageal tumor” [MeSH] or “esophageal tumors”[MeSH] or “Esophagus tumors” [MeSH] or “Esophagus tumor” [MeSH] or “esophageal squamous cell carcinoma” [MeSH] or “ESCC” [MeSH] or “EAC” [All Fields] or “esophageal squamous carcinoma” [MeSH] or “esophageal adenocarcinoma” [MeSH] or “adenocarcinoma of the esophagus” [MeSH]) and (“Helicobacter pylori” [MeSH] or “H pylori” [MeSH] or “H. pylori” [MeSH] or “HP” [All Fields] or “Helicobacter” [MeSH]).

Embase search terms:

(“Esophageal Neoplasm” [Emtree] or “Neoplasm, Esophageal” [Emtree] or “Esophagus Neoplasm” [Emtree] or “Esophagus Neoplasms” [Emtree] or “Neoplasm, Esophagus” [Emtree] or “Neoplasms, Esophagus” [Emtree] or “Neoplasms, Esophageal” [Emtree] or “Esophageal Neoplasms” [Emtree] or “Cancer of Esophagus” [Emtree] or “Cancer of the Esophagus” [Emtree] or “Esophagus Cancer” [Emtree] or “Cancer, Esophagus” [Emtree] or “Cancers, Esophagus” [Emtree] or “Esophagus Cancers” [Emtree] or “Esophageal Cancer” [Emtree] or “Cancer, Esophageal” [Emtree] or “Cancers, Esophageal” [Emtree] or “Esophageal Cancers” [Emtree] or “esophageal carcinoma” [Emtree] or “esophageal carcinomas” [Emtree] or “Esophagus carcinoma” [Emtree] or “Esophagus carcinomas” [Emtree] or “esophageal tumor” [Emtree] or “esophageal tumors” [Emtree] or “Esophagus tumors” [Emtree] or “Esophagus tumor” [Emtree] or “esophageal squamous cell carcinoma” [Emtree] or “ESCC” [Emtree] or “EAC” [Emtree] or “esophageal squamous carcinoma” [Emtree] or “esophageal adenocarcinoma” [Emtree] or “adenocarcinoma of the esophagus” [Emtree]) and (“Helicobacter pylori” [Emtree] or “H pylori” [Emtree] or “H. pylori” [Emtree] or “HP” [Emtree] or “Helicobacter” [Emtree]).
